# Out‐of‐pocket costs associated with head and neck cancer treatment

**DOI:** 10.1002/cnr2.1528

**Published:** 2021-08-24

**Authors:** Mohemmed N. Khan, Katrina Hueniken, Mirko Manojlovic‐Kolarski, Lawson Eng, Maryam Mirshams, Khaleeq Khan, Colleen Simpson, Michael Au, Geoffrey Liu, Wei Xu, Christopher J. Longo, David P. Goldstein, Jolie Ringash, Rosemary Martino, Aaron R. Hansen, John R. de Almeida

**Affiliations:** ^1^ Department of Otolaryngology ‐ Head and Neck Surgery Mount Sinai Medical Center New York New York USA; ^2^ Department of Biostatistics Princess Margaret Cancer Centre Toronto Canada; ^3^ Department of Otolaryngology – Head and Neck Surgery Princess Margaret Cancer Centre Toronto Canada; ^4^ Department of Medical Oncology and Hematology Princess Margaret Cancer Centre Toronto Canada; ^5^ Department of Health Policy and Management McMaster University Hamilton Canada; ^6^ Department of Radiation Oncology Princess Margaret Cancer Centre Toronto Canada; ^7^ Institute of Health Policy Management and Evaluation University of Toronto Toronto Canada; ^8^ Department of Speech Language Pathology University of Toronto Toronto Canada

**Keywords:** chemotherapy, head and neck cancer, out‐of‐pocket costs, quality of life, radiation, surgery

## Abstract

**Background:**

Out‐of‐pocket costs (OOPC) associated with treatment have significant implications on quality of life and survival in cancer patients. Head and neck cancer patients face unique treatment‐related challenges, but to date OOPC have been understudied in this population.

**Aims:**

This study aims to identify and measure OOPC for patients with head and neck cancer (HNC) in Ontario.

**Methods:**

HNC patients between 2015 and 2018 at Princess Margaret Cancer Centre in Toronto were recruited. Participants completed OOPC questionnaires and lost income questions during radiation, post‐surgery, and 3, 6, 12, and 24 months after completion of treatment. Associations between OOPC and treatment modality and disease site were tested with multivariable hurdle regression.

**Results:**

A total of 1545 questionnaires were completed by 657 patients. Median estimated OOPC for the total duration of treatment for participants undergoing chemoradiation was $1452 [$0–14 616], for surgery with adjuvant radiation or chemoradiation (C/RT) was $1626, for radiation therapy alone was $635, and for surgery alone was $360. The major expenses for participants at the mid‐treatment time‐point was travel (mean $424, standard error of the mean [SEM] $34) and meals, parking, and accommodations (mean $617, SEM $67). In multivariable analysis, chemoradiation, surgery with C/RT, and radiation were associated with significantly higher OOPC than surgery alone during treatment (791% higher, *p* < .001; 539% higher, *p* < .001; 370% higher, *p* < .001 respectively) among patients with non‐zero OOPC. Participants with non‐zero OOPC in the laryngeal cancer group paid 49% lower OOPC than those with oropharyngeal cancers in adjusted analysis (*p* = .025).

**Conclusions:**

Patients undergoing treatment for HNC pay significant OOPC. These costs are highest during treatment and gradually decrease over time. OOPC vary by patient demographics, clinical factors, and, in particular, treatment modality.

## 1 INTRODUCTION

The costs associated with cancer care may be significant and are often overlooked by healthcare providers.^1^, ^2^ Out‐of‐pocket costs (OOPC) for medical care may cause distress among cancer patients and their families and impact their social, psychological, and spiritual well‐being.^3^ The OOPC borne by cancer patients may impact their ability to participate in treatment and surveillance, which may in turn affect their prognosis.^4^ New treatment and surveillance modalities may lead to increased OOPC.^5^, ^6^

In Canada and in the province of Ontario, healthcare coverage is universal. However, many costs are still incurred by patients including travel and accommodation costs, prescription medications, and many allied health care services. Some of these costs may be offset for patients who have private insurance coverage. Despite having universal healthcare coverage, patients may suffer a significant financial burden for cancer care.^7^, ^8^

Recent studies have shown that cancer patients are more likely to forego medical care or medications due to financial concerns than other patients.^9^, ^10^ Moreover, as many as 13.4% of cancer patients spend more than 20% of their income on health care.^11^ Annual medical expenditure and productivity loss have been investigated in specific types of cancer, such as breast, colorectal, and prostate cancer.^12^, ^13^, ^14^ Although there have been some studies describing the magnitude of OOPC in head and neck cancer (HNC) patients in countries such as China and India,^15^, ^16^, ^17^, ^18^, ^19^ there is little work to date that has investigated the impact of OOPC in HNC patients in North America.^20^, ^21^ Prior North American studies have utilized national databases, such as the Surveillance Epidemiology and End Results (SEER), to estimate OOPC.^22^, ^23^, ^24^ This approach to estimating OOPC may not fully capture all relevant costs or the full financial impact on patients. The Patient Self‐Administered Financial Effects (P‐SAFE) instrument was previously designed to examine the financial effects of a cancer diagnosis.^7^, ^25^ However, we felt the OOPC associated with HNCs are unique and as such we developed and validated a new tool to examine the costs in this specific population. To our knowledge, this is the first study aiming to comprehensively measure different types of OOPC incurred by HNC patients in North America.

## 2 METHODS

### 2.1 Study population and data sources

From 2015 to 2018, adult (>18) patients with HNCs (oral cavity, pharynx, larynx, nasal cavity, and unknown primaries) treated with curative intent were approached to participate in a prospective study at the Princess Margaret Cancer Centre in Toronto, Ontario, Canada. Ethics approval was obtained for this study from the University Health Network (UHN) Research Ethics Board (REB), REB#07‐0521. Informed consent was obtained from each participant. Participants' OOPC were assessed immediately after surgery (in surgically resected patients); at mid‐radiation treatment (in patients receiving radiation treatment); and at 3, 6, 12, and 24 months after completion of treatment. The questionnaire was administered prospectively to all potentially interested patients. Patients were enrolled from all timepoints simultaneously. As such, not all patients were followed from treatment initiation to the last follow‐up; some were enrolled well after their treatment. Demographic data and household income were collected around the time of diagnosis; patient‐reported lost wages due to cancer was collected at 12 months post‐treatment.

The OOPC questionnaire used in this study (Figure S1) was developed de novo at the inception of the study. Literature review was conducted to identify salient areas of out‐of‐pocket expenditure for HNC patients. MEDLINE was searched for medical subject headings and keywords relating to out‐of‐pocket costs, personal expenditures and HNC, up to December 2013. Categories of spending identified by literature search were included in the instrument. The questionnaire was pilot‐tested and expert reviewed prior to administration.

Dollar values were reported for OOPC related to travel, meals, parking, accommodations, homecare, child care, and domestic assistance. Separate questionnaire modules were included for patients to report individual item costs for medical supplemental expenses, as well as ancillary care costs not covered by governmental or private health insurance (i.e., dental, physiotherapy, nutritionist, etc.). All costs were reported in Canadian dollars (CAD).

All reported OOPC were summed to create a single estimated dollar value for OOPC incurred during the month prior to the administration of the questionnaire. For items where no dollar value was reported, OOPC was assumed to be $0 for that item. For patients undergoing surgery and adjuvant radiation or chemoradiation (C/RT), the costs from the post‐operative administration as well as the mid‐radiation therapy questionnaire were summed as an estimate of “on treatment OOPC.” For surgically treated patients, the duration of treatment was considered to be 1 month, to account for recovery time. To estimate OOPC over the duration of radiation therapy with or without concurrent chemotherapy, the OOPC reported during the mid‐treatment administration were multiplied by the total duration of treatment in months. For example, if a patient had surgery plus 6 weeks of adjuvant radiation, then that patient's total estimated mid‐treatment cost is the sum of their post‐surgery OOPC in the past month (multiplied by 1, for one‐month duration of treatment) plus their reported mid‐radiation OOPC in the past month (multiplied by 42/30.44 days, or 1.38‐month duration of radiation). Nasopharyngeal cancer patients who continued to be treated with adjuvant chemotherapy after radiation/chemoradiation were removed from estimated treatment cost analyses, as costs during chemotherapy were not captured.

Household income and lost income due to cancer were categorized into income ranges by dollar values in $40 000 increments from $0 to $39 999, then $40 000 to $79 999, and over $80 000, all in Canadian dollars. Relative lost household income was computed by dividing lost household income by baseline pre‐treatment household income, to indicate the percent of baseline income lost due to cancer.

### 2.2 Statistical analysis

#### 2.2.1 Instrument validation

We tested the following a priori hypotheses: (1) the number of reported trips to the hospital should correlate with OOPC, travel costs, and travel‐related costs (total meals, parking, and accommodation), and (2) patients reporting car and taxi travel will have higher travel costs. These hypotheses were testing using Spearman rank correlations. Mild correlations were defined by rho = ±0.15–0.39, moderate by rho = ±0.4–0.59, and strong correlations by rho ±0.6–1.0.

#### 2.2.2 Analysis

The distribution of OOPC across time‐points was reported descriptively. Baseline household income and lost income due to cancer are reported using descriptive statistics. Spearman correlations were computed between OOPC and baseline income, as well as OOPC and lost income due to cancer. Kruskal–Wallis tests were used to determine associations between treatment modality and lost income due to cancer. The association between lost income and OOPC was assessed by Spearman correlation.

The trajectory of OOPC over time in each treatment group was plotted using a line graph, with error bars to represent 95% confidence intervals on the mean OOPC in each treatment group, at each time‐point. Mean OOPC at the “Mid‐treatment” time‐point was taken as the mean estimated cost over the course of treatment (combining post‐surgery and mid‐radiation for patients treated with multi‐model therapy); OOPC at all other time‐points are reported for the prior month.

Univariable and multivariable regression analyses were used to test associations between OOPC and clinico‐demographic patient characteristics. Regression analysis was conducted for both mid‐treatment costs (using total estimated OOPC during treatment) and follow‐up costs at 3 months post‐treatment. As is often the case with studies measuring cost data, the distribution of total OOPC was right‐skewed and zero‐inflated (a significant proportion of patients reported zero costs). We therefore applied a hurdle model, a two‐part mixture model that accounts for two separate stochastic processes occurring within the data. First, the probability of patients reporting non‐zero OOPC was modeled using logistic regression. Then, among patients reporting non‐zero costs, OOPC were log‐transformed and modeled using linear regression. This log‐linear regression was used to find the percent increase in OOPC attributable to group membership for each clinico‐demographic factor. Variable selection for multivariable models was conducted by keeping any variable with *p* < .1 in the logistic or linear regression steps.

## 3 RESULTS

### 3.1 Patients

A total of 657 patients completed a total of 1545 questionnaires included in the study. Demographic information is reported in Table 1. Survey compliance, or the proportion of eligible patients who completed OOPC questionnaires at each time point, is reported in Table 2. Among participants who reported their household income prior to their cancer diagnosis, 51% of participants had household income of at least $60 000 and 21% of participants had household income greater than $100 000. Almost all patients had provincial insurance (*N* = 636, 97%) and half of the patients had extended health insurance (*N* = 333, 51%). The majority of patients (*N* = 413, 63%) had an additional drug plan.

**TABLE 1:** Patient demographics

Total *N* = 657	Overall *N* (%)
Age at recruitment, years median [range]	62.5 [22.7–92.3]
*Gender*	
Female	149 (22.7)
Male	508 (77.3)
*Marital status*	
Not married	164 (26.0)
Married/common‐law	457 (74.0)
*Unknown*	*36*
*Ethnicity*	
Non‐white	98 (18.0)
White/Caucasian	447 (82.0)
*Unknown*	*112*
*Highest education*	
College/University/Professional School	261 (48.1)
Vocational/Technical School	36 (6.6)
High School Graduate	140 (25.8)
Less than High School Grad	105 (19.4)
*Unknown*	*115*
*Household income*	
<$39 999	101 (29.9)
40 000−79 999	109 (32.2)
$80 000+	128 (37.9)
*Missing/prefer not to say*	*319*
*Clinical stage*	
I	106 (16.3)
II	72 (11.1)
III	97 (14.9)
IV	375 (57.7)
*Unknown*	*7*
Disease site	
Oropharynx	304 (46.3)
Lip and oral cavity	144 (21.9)
Larynx	99 (15.1)
Nasopharynx	42 (6.4)
Hypopharynx	19 (2.9)
Nasal cavity	11 (1.7)
Other/unknown primary	38 (5.8)
*Treatment modality*	
Chemoradiation	235 (35.8)
Surgery + (chemo)radiation	86 (13.1)
Radiation	240 (36.5)
Surgery only	96 (14.6)

**TABLE 2:** Out‐of‐pocket costs in the past month, by time point

	Total # of eligible patients for OOPC questionnaire completion^a^	# of patients who completed questionnaire (included in analysis)	Proportion of eligible patients who completed questionnaire	Median per‐month OOPC (CAD)	Median per‐month OOPC for Pts who paid >$0 CAD	Range of per‐month OOPC For Pts who paid >$0 CAD
Any time point, *N*	952	657	69%	NA	NA	NA
Post‐surgery, pre‐adjuvant treatment	211	111	53%	350	400	20–5350
Mid‐radiation	553	320	58%	619	725	10–7915
3 months	706	387	55%	150	195	6–11 250
6 month	639	236	37%	70	100	5–10 312
12 month	823	279	34%	40	83	3–5110
24 month	529	212	40%	40	80	2–5040

Abbreviations: NA, not applicable; OOPC, out‐of‐pocket cost.

^a^ Total # eligible patients includes all patients who, at time of study entry, were eligible to complete questionnaires at each time point. Includes patients who passed away or were lost to follow‐up between study entry and follow‐up time points.

### 3.2 Questionnaire validation

Questionnaire validation was performed on all patients across time points. Number of self‐reported trips to the cancer center were mildly correlated with OOPC in the past month (rho = 0.33, *p* < .001), travel costs (rho = 0.26, *p* < .001), and total meals, parking, and accommodation costs (rho = 0.18, *p* < .001). Patients who reported using a car or taxi to travel to the hospital had higher travel costs than those who did not (median $50 vs. $20; *p* < .001), while patients who used public transit had lower travel costs than those who did not (median $20 vs. $50, *p =* .011).

### 3.3 Out‐of‐pocket costs

The median OOPC paid in the month prior to survey administration was $350 [range $0–5350] for the post‐surgery, pre‐adjuvant treatment survey. Median OOPC in the past month for participants undergoing radiation was $619 [$0–7915]. Median OOPC for all patients during post treatment follow‐up was $150 [$0–11 250] at 3 months post‐treatment, $70 [$0–10 312] at 6 months, $40 at 12 months, and $40 [$0–5040] at 24 months (Table 2).

**FIGURE 1:**
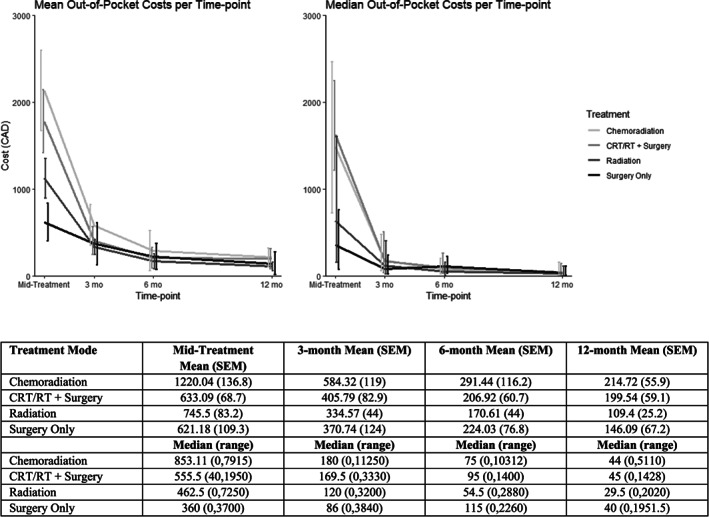
Mean and median out‐of‐pocket costs over time, stratified by treatment modality

Median duration of treatment was 49 days [range 20–95 days]. Median estimated OOPC for the total duration of treatment for patients undergoing chemoradiation therapy was $1452 [range $0–14 616] with a median treatment duration of 55 days; for those undergoing surgery with adjuvant treatment was $1626 [$121–4905] (median duration 81 days), for those undergoing radiation therapy alone was $635 [$0–8099] (median duration 48 days), and for those undergoing surgery alone was $360 [$0–3700] (median duration 30 days). Mean OOPC in each treatment group from treatment to 12‐month follow‐up are presented in Figure 1. Figure S3 shows mean and median OOPC over time for other clinico‐demographic factors.

OOPC were highest during treatment in each group and decreased over the course of post‐treatment follow‐up. The most commonly reported medical supplemental cost during treatment was prescription or over‐the‐counter medication. One hundred and twenty‐eight patients (28.1%) reported medical supplemental expenditures greater than zero, with median medical supplemental costs of $80 (IQR $50–150). After surgery, 31 patients (7.2%) spent greater than $0 on medication (median cost = $50, IQR $22.50–100). The most commonly reported ancillary care expense was dental visits; 101 radiation patients (22.2%) spent greater than $0 on dental care with a median OOPC of $300 (IQR $100–485). Few surgical patients reported dental expenses after surgery (*N* = 4 with OOPC >$0; median $112.50, IQR $90–143.75).

**FIGURE 2:**
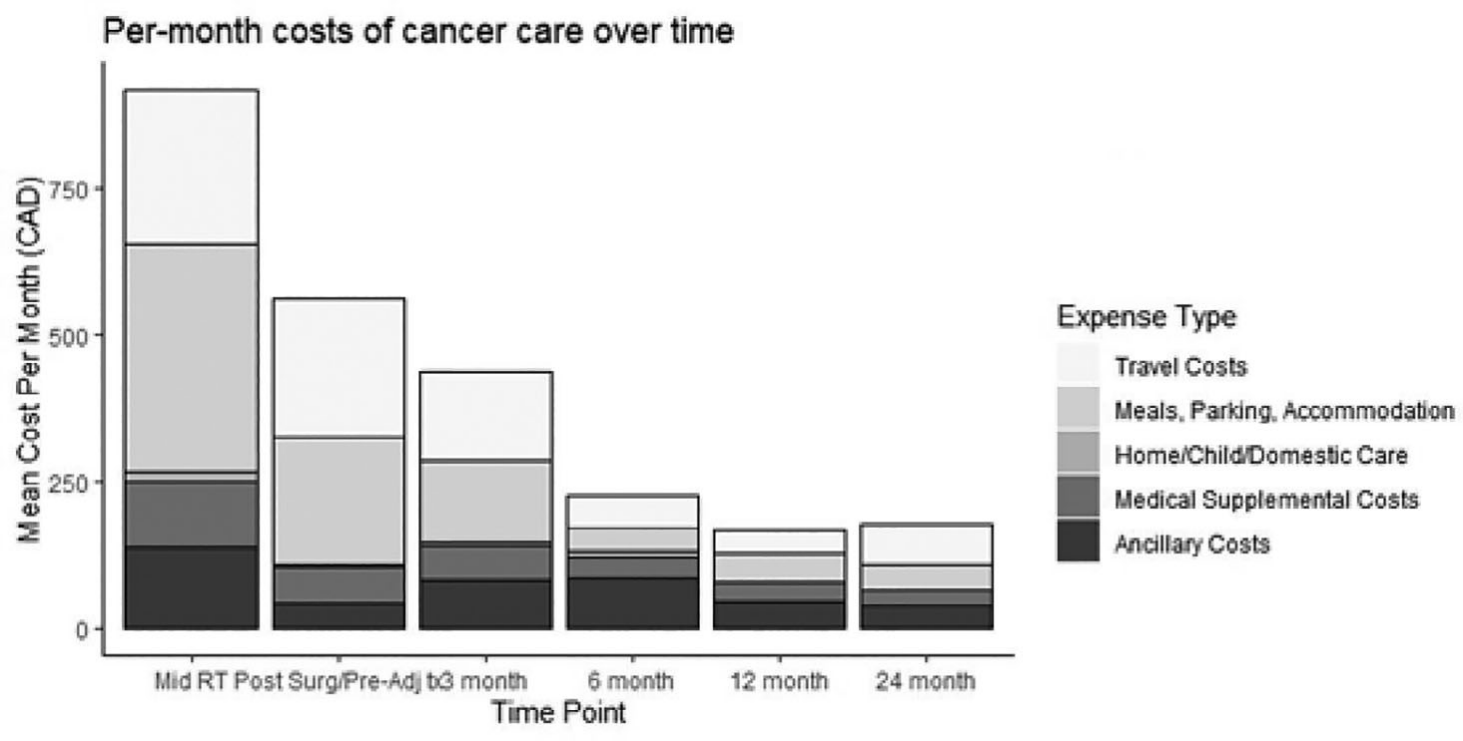
Total out‐of‐pocket costs over time

The distribution of individual costs over the past month for each time‐point is shown in Figure 2. Travel, meals, parking, and accommodation make up the majority of OOPC at each time‐point. Home care, child care, and domestic care costs greater than zero were reported by only 28 out of 657 patients throughout the study, and made the smallest contribution to mean OOPC. Estimated mid‐treatment cost was $424 for travel (standard error of the mean [SEM] $34); $617 (SEM $67) for meals, parking, and accommodation; $24 (SEM $10) for home, child, or domestic care; $180 (SEM $20) for medical supplemental costs; and $211 (SEM $23) for ancillary service costs. Median lost wages due to cancer care reported at 12 months post‐treatment among patients under 65 years of age (*N* = 98) was $25 000 CAD (IQR $5000–45 000). Relative to baseline pre‐cancer household income, patients under age 65 reported having lost a median of 20% of their income due to cancer at 12‐month follow‐up (IQR: 6–45% of baseline income). For patients age 65 years and over (*N* = 40), median lost income was $5000 (IQR $5000–17 500) or 11% of baseline income (IQR 5–28%). Among patient under 65 years, lost income at 12 months was not significantly correlated with treatment modality (*p =* .33) or OOPC during treatment (*p* = .08).

### 3.4 Relationships between OOPC and clinico‐demographic factors

Results of univariable and multivariable regression analysis on estimated OOPC during treatment are presented in Table 3. 91% of patients reported OOPC greater than $0 overall. Treatment modality, stage at diagnosis, ECOG status, disease site, employment, education, income, and age were retained in the multivariable model after variable selection. Among those with greater than $0 OOPC, treatment modality remained significant in adjusted analyses; participants receiving radiation paid 370% more than those with surgery alone during treatment (*p* < .001). The group with surgery plus C/RT paid 539% more (*p* < .001), and the chemoradiation group paid 791% more (*P* < .001). Participants with non‐zero OOPC in the laryngeal cancer group paid 49% lower OOPC than those with oropharyngeal cancers in adjusted analysis (*p* = .025).

**TABLE 3:** Univariable and Multivariable regression analysis on OOPC during treatment (*N* = 329)

	Univariable				Multivariable			
	Percent non‐zero OOPC	*p*‐Value	Percent increase in costs (OOPC >$0)^a^	*p*‐Value	Odds ratio for non‐zero OOPC^b^	*p*‐Value	Percent increase in costs (OOPC >$0)^a^	*p*‐Value
*Treatment modality*								
Surgery alone	88%	Ref	Ref	Ref	Ref	Ref	Ref	Ref
Radiation	88%	0.993	79%	0.005	0.68	0.835	370%	<0.001
Surgery + C/RT	100%	0.988	285%	<0.001	‐	0.992	539%	<0.001
Chemoradiation	92%	0.398	286%	<0.001	0.62	0.804	791%	<0.001
*Gender*								
Female	89%	Ref	Ref	Ref	NA	NA	NA	NA
Male	91%	0.508	‐7%	0.680	NA	NA	NA	NA
*Stage*								
Early (I‐III)	92%	Ref	Ref	Ref	Ref	Ref	Ref	Ref
Late (IV)	90%	0.599	58%	0.003	0.84	0.828	−3%	0.760
*ECOG*								
0	93%	Ref	Ref	Ref	Ref	Ref	Ref	Ref
1	88%	0.126	−15%	0.299	1.48	0.535	−16%	0.353
2+	82%	0.18	−67%	0.012	0.80	0.865	−50%	0.242
*Disease site*								
Oropharynx	91%	Ref	Ref	Ref	Ref	Ref	Ref	Ref
Lip and oral cavity	90%	0.789	−40%	0.006	1.15	0.939	42%	0.406
Larynx	91%	0.942	−47%	0.004	1.49	0.711	−49%	0.025
Nasopharynx	89%	0.834	−14%	0.743	0.60	0.692	−56%	0.115
Other/unknown primary	93%	0.742	−32%	0.144	1.99	0.554	−34%	0.195
*Employment status*								
Employed	93%	Ref	Ref	Ref	Ref	Ref	Ref	Ref
Not employed	91%	0.666	−34%	0.01	1.73	0.445	−10%	0.586
Highest education								
College/University/Professional School	93%	Ref	Ref	Ref	Ref	Ref	Ref	Ref
Vocational/Technical School	90%	0.657	−32%	0.215	0.41	0.361	−33%	0.270
High School Graduate	94%	0.78	5%	0.808	1.00	0.997	−18%	0.356
Less than High School Grad	84%	0.05	−16%	0.424	0.67	0.590	−9%	0.696
*Household income*								
<$39 999	87%	Ref	Ref	Ref	Ref	Ref	Ref	Ref
$40000‐79 999	96%	0.065	18%	0.473	3.49	0.096	−3%	0.894
$80 000+	95%	0.088	35%	0.190	2.23	0.269	−9%	0.680
*Marital status*								
Married/common‐law	92%	Ref	Ref	Ref	NA	NA	NA	NA
Divorced/Separated/Widowed	91%	0.839	−21%	0.254	NA	NA	NA	NA
Never Married	88%	0.469	−5%	0.861	NA	NA	NA	NA
Age (10 year increments)	91%	0.107	−20%	0.004	0.51	0.110	−20%	0.323

*Note*: Data were modeled using a two‐part hurdle model.

Abbreviations: NA, not applicable; OOPC, out‐of‐pocket cost; Ref, reference value.

^a^ Coefficients in this column represent the increase in OOPC (or decrease, if negative) for each group for participants who paid >$0 only, compared to the indicated reference category. The coefficient for age represents the % change in OOPC associated with a 10‐year increase in age.

^b^ Odds ratios in this column represent the change in odds of reporting non‐zero OOPC for each group of participants, compared to the reference category. The OR for age represents the change in odds of reporting non‐zero OOPC associated with a 10‐year increase in age.

Three hundred and eighty‐four patients contributed OOPC at 3‐months post‐treatment; of those, 85% of patients reported costs greater than zero. Univariable and multivariable analysis is presented in Table 4. For the 3‐month post‐treatment multivariable model, treatment modality, disease site, and education were retained after variable selection. In multivariable analysis, treatment modality was not significantly associated with the odds of paying any OOPC, or amount paid above $0. Participants who paid greater than $0 with laryngeal cancers also had significantly lower odds of reporting non‐zero costs compared to oropharynx (*p* = .001).

**TABLE 4:** Univariable and Multivariable regression analysis on OOPC at 3 month post‐treatment follow‐up (*N* = 384)

	Univariable				Multivariable			
	Percent non‐zero OOPC	*p*‐Value	Percent increase in costs (OOPC >$0)^a^	*p*‐Value	Odds ratio for non‐zero OOPC^b^	*p*‐Value	Percent increase in costs (OOPC >$0)^a^	*p*‐Value
*Treatment modality*								
Surgery Alone	88%	Ref	Ref	Ref	Ref	Ref	Ref	Ref
Radiation	81%	0.271	56%	0.102	1.24	0.784	70%	0.218
Surgery + C/RT	82%	0.395	101%	0.03	0.95	0.941	87%	0.087
Chemoradiation	91%	0.684	101%	0.01	2.44	0.284	107%	0.089
*Gender*								
Female	88%	NA	Ref	NA	NA	NA	NA	NA
Male	85%	0.529	15%	0.452	NA	NA	NA	NA
*Stage*								
Early (I‐III)	82%	Ref	Ref	Ref	NA	NA	NA	NA
Late (IV)	87%	0.175	11%	0.523	NA	NA	NA	NA
*ECOG*								
0	88%	Ref	Ref	Ref	NA	NA	NA	NA
1	83%	0.179	28%	0.139	NA	NA	NA	NA
2+	78%	0.392	−19%	0.709	NA	NA	NA	NA
*Disease site*								
Oropharynx	91%	Ref	Ref	Ref	Ref	Ref	Ref	Ref
Lip and oral cavity	85%	0.162	−18%	0.333	0.74	0.687	−1%	0.988
Larynx	70%	<0.001	−45%	0.02	0.19	0.001	−33%	0.196
Nasopharynx	86%	0.513	18%	0.645	0.47	0.371	−3%	0.937
Other/unknown primary	83%	0.165	−42%	0.046	0.48	0.243	−35%	0.163
*Employment status*								
Employed	90%	Ref	Ref	Ref	NA	NA	NA	NA
Not employed	84%	0.136	22%	0.251	NA	NA	NA	NA
*Highest education*								
College/University/Professional School	91%	Ref	Ref	Ref	Ref	Ref	Ref	Ref
Vocational/Technical School	85%	0.445	−37%	0.231	0.71	0.633	−32%	0.304
High School Graduate	83%	0.095	−6%	0.77	0.5	0.108	−9%	0.687
Less than High School Grad	84%	0.198	−13%	0.548	0.78	0.59	−8%	0.740
*Household income*								
<$39 999	82%	Ref	Ref	Ref	NA	NA	NA	NA
$40000–79 999	90%	0.165	−6%	0.793	NA	NA	NA	NA
$80 000+	89%	0.212	16%	0.535	NA	NA	NA	NA
*Marital status*								
Married/common‐law	88%	Ref	Ref	Ref	NA	NA	NA	NA
Divorced/Separated/Widowed	86%	0.575	−5%	0.828	NA	NA	NA	NA
Never Married	79%	0.176	−29%	0.298	NA	NA	NA	NA
Age (10 year increments)	91%	0.168	1%	0.937	NA	NA	NA	NA

Abbreviations: NA, not applicable; OOPC, out‐of‐pocket cost; Ref, reference value.

^a^ Coefficients in this column represent the increase in OOPC (or decrease, if negative) for each group for participants who paid >$0 only, compared to the indicated reference category. The coefficient for age represents the % change in OOPC associated with a 10‐year increase in age.

^b^ Odds ratios in this column represent the change in odds of reporting non‐zero OOPC for each group of participants, compared to the reference category. The OR for age represents the change in odds of reporting non‐zero OOPC associated with a 10‐year increase in age.

Due to the high proportion of participants who did not report household income, we performed a sensitivity analysis while including those who did not indicate an income. In one analysis, this group had a higher likelihood of non‐zero OOPC than those with income <$40 000, however this was not statistically significant. After including those who did not indicate an income the results of multivariable analysis were in keeping with the original analysis and the predictors of OOPC in the original analysis were maintained.

Subgroup analysis was performed to examine the differences between disease sites. A higher proportion of larynx cancer patients were treated with radiation alone (80.8 vs. 42.1%) and had early stage 0‐II disease (62.7 vs. 5.9%) compared to the reference oropharynx group. When we assessed specific costs by disease site (Figure S2) oropharynx cancer had higher costs in all categories with the exception of home, child, and domestic care which also had the lowest contribution to overall OOPCs.

## 4 DISCUSSION

A diagnosis of cancer is often unexpected, and while patients attempt to cope with the emotional aspects of their diagnosis as well as their own mortality, they may be underprepared for the financial repercussions of cancer treatment and side‐effects. Out‐of‐pocket cancer‐related costs have been understudied in HNCs, particularly in single‐payer healthcare systems where major costs of treatment are often assumed to be covered by government insurance. Therefore, it may be difficult for health care providers to recognize and refer patients who are at risk of significant financial burden. HNCs are associated with the most significant symptom burden following treatment of all common cancers and these toxicities may add additional financial burden for patients such as the need for additional prescription medications or supplies; consultations for services for which the patient may bear some or all of the costs such as outpatient dentistry, physiotherapy, or swallowing therapy; and costs for travel for health care.^26^ Our study aimed to examine the extent of OOPC incurred by HNC patients in Canada. Furthermore, we analyzed the relative contributions of individual costs that make up patients' overall OOPC, and the variation in OOPC between treatment modalities and over time from treatment.

As expected, patients undergoing chemoradiotherapy or surgery with C/RT, and those with advanced‐stage disease, incurred the greatest OOPC during treatment. Patients treated with surgery alone had the lowest OOPC; this may be due to reduced need for frequent travel, and fewer additional medical expenses associated with prolonged treatment. Our findings were consistent with those of another Canadian study by Longo et al, which showed that cancer patients receiving chemotherapy had higher overall OOPC than those without chemotherapy.^27^ Prior studies have also shown a tendency toward greater expenditure and financial hardship in patients with advanced stage disease when compared with early stage disease counterparts.^28^, ^29^ The difference in OOPC within the larynx cancer site is likely due to the treatment modality (radiation alone versus multimodality) and earlier stage of diagnosis were likely drivers of the lower OOPCs in the larynx cancer disease site. There did not appear to be a single element driving the lower OOPCs.

In our study, the largest expense incurred by patients, regardless of treatment modality, was travel and travel‐related costs (meals, parking, and accommodations). Patients undergoing radiation therapy, whether alone or in conjunction with other treatment modalities, incur significant travel and accommodation expenses due to the need for daily treatment visits during the week. It should be noted that the delivery of radiotherapy for HNC patients is centralized in Ontario due to the need for highly specialized resources. Due to this specialization, treatment usually requires travel to higher volume centers often located in busy cities.^30^

Patients' costs tended to be highest during treatment and decrease as they completed treatment and began surveillance. An increase in the number of trips needed for cancer related services may be a contributing factor to increased OOPC. Recommendations for recurrence screening for HNC patients requires less frequent appointments as time passes, requiring less travel and OOPC on the part of the patient.^31^ In our study, ancillary medical services and medical supplemental costs tended to take up a larger proportion of total OOPC in the post treatment period. For patients who required ancillary services, such as dental services, speech therapy, and physical therapy, these expenses became a major source of OOPC during the surveillance period. Cancer survivorship and post‐treatment needs can be significant in head and neck patients specifically due to the complex nature of the anatomy and functional implications of treatment in this area. Many patients require speech or swallowing assistance, dental rehabilitation/treatment, physical therapy, nutritional support, and occupational therapy.^32^, ^33^

This study has several limitations. First, as participants are asked to respond to a questionnaire, the data is susceptible to recall bias. However, this questionnaire was designed to capture costs only over the previous month, to minimize recall bias as much as possible. Despite our attempts to control for recall bias, it did appear to factor into the mild correlation with pre‐selected variables in our de novo questionnaire. We do note, however, that unlike traditional patient reported outcomes and quality of life instruments, our questionnaire does not lend itself well to validation testing due to its quantitative nature. Second, results may be subject to volunteer bias: participants who agreed to participate in the study may be systematically different than those that did not as patients' ability and interest in participating may correlate with demographic or clinical characteristics. Third, assumptions used to model the calculated average OOPC at each time point might be incorrect: missing responses to individual cost questions may not have been intended by patients to indicate zero cost; and OOPC do not necessarily remain constant over the course of treatment. Fourth, patients treated in Ontario, Canada, may have a different OOPC experience than those in jurisdictions without universal health care. While other factors including younger age,^13^, ^34^, ^35^ lower household income,^11^, ^36^, ^37^ and lower education level^11^, ^37^ have previously been associated with higher costs in our jurisdictions, these were not independently significant in our study. This is in keeping with other published literature from Canada that has shown income and education level to be poor predictors of OOPC.^27^ This likely reflects the nature of head and neck cancer care in Ontario, which is universal and centralized. Fifth, 48.5% of patients preferred not to report their income, which limited ability to interpret the overall burden of OOPC on this sample. We attempted to address this by performing a sensitivity analysis that included this group, and were able to confirm our initial findings. Lastly, collecting patients' perceptions of their OOPC was beyond the scope of this study. This is important to consider as objective costs may not fully reflect the impact of treatment. The financial toxicity of cancer treatment is being increasingly recognized and there are specific instruments to evaluate for it including the COmprehensive Score for financial Toxicity (COST) and Financial Index of Toxicity (FIT) instruments, the latter which was developed specifically for HNC.^38^, ^39^ Additional research on the perceived impact of OOPC on patients' finances, willingness to pay additional medical costs, and patients' level of regret about making medical decisions due to costs, would be an important component in assessing the true burden of cancer‐related OOPC.

In conclusion, this study found that Canadian HNC patients pay significant OOPC toward their cancer care, which tend to be highest during treatment and decrease over time. These costs vary by patient demographics, clinical factors, and, in particular, treatment modality.

## CONFLICT OF INTEREST

The authors declare there is no conflict of interest.

## AUTHOR CONTRIBUTIONS

*Study conceptulaization and Formal Analysis*, M.N.K., K.H., W.X., and J.D.A.; *Funding Acquisition*, *Methodology*, J.D.A.; M.M.K., M.A., L.E., G.L., and C.J.L.; *Data Aquisition*, M.M. and K.K.; *Data Curation*, C.S.; *Data Validation*, K.H. and J.D.A.; *Writing of the Original Manuscript*, M.N.K.; *Review and Editing*, M.M.K. and M.A.; *Tables and Figures*, M.N.K., M.M.K. and K.H.; *Supervisors*, C.J.L., D.P.G., J.R., R.M., and A.R.H.

## ETHICAL STATEMENT

Ethics approval was obtained for this study from University Health Network (UHN) Research Ethics Board (REB), REB#07‐0521. Informed consent was obtained from each participant.

## Supporting information


**Figure S1** Out‐of‐Pocket Cost Questionnaire

**Figure S2** Individual Out of Pocket Costs by Disease Site

**Figure S3** Mean and Median Out‐of‐Pocket Costs over Time

## Data Availability

Authors elect to not share data.
