# Supplementary material for: Out‐of‐pocket costs associated with head and neck cancer treatment
Source: Cancer Rep (Hoboken). 2021 Aug 24;5(7):e1528. doi: 10.1002/cnr2.1528 (PMC9327650; doi:10.1002/cnr2.1528)
Supplement: Supplementary file 1 — Figure S1 Out‐of‐Pocket Cost Questionnaire [file CNR2-5-e1528-s001.docx]

**Supplementary Figure 1: Out-of-Pocket Cost Questionnaire**

**Follow-up Questionnaire**

**Date: _________________________**

Please answer the following questions about your health now and about some of your health habits. This information will be kept strictly confidential, and will only be shared with members of your treatment team and used for medical research purposes as specified on the consent form.

**Part A: Out of Pocket Expenses**

Note: Out-of-pocket costs refer to costs related to your cancer treatment for which you have had to pay directly. In some instances, these will have been covered by an insurance policy or drug plan. Please indicate the following relevant costs/expenses and where you have had to pay directly or (where indicated) you have had these costs covered by insurance or drug plans.

| 1. | Do you have OHIP coverage? | ☐ No | ☐ Yes |
| --- | --- | --- | --- |
| 2. | Do you have an extended health insurance policy? | ☐ No | ☐ Yes |
|  | a. If so, which insurance policy do you have: _______________________________ | | |
| 3. | Do you have a drug plan? | ☐ No | ☐ Yes |

- 1. If so, which plan: ___________________________________________________

1. Do you receive any additional financial assistance (e .g. governmental assistance, charitable assistance) to cover your out-of-pocket medical costs?

☐ No ☐ Yes

If so, how much do you receive monthly? ($)_________________________________________

What is the source of funding? ____________________________________________________

**TRAVEL/ACCOMODATIONS COSTS**

For questions 5 – 8, please indicate in **the past month** how much did you spend out-of-pocket ($) on the following (specifically related to your cancer treatment)

1. a. Travel (gas/bus fare/train fare/air fare): ($)________________________________________
   1. Do you: ☐ Travel from home ☐ Stay at the Princess Margaret Lodge ☐ Stay at an alternative accommodation, please indicate where: ___________________________________

1. What is the first 3 letters of the postal code of the place at which you currently stay? __________
2. What mode of transportation do you most often use to get to the hospital/clinic? _________
3. How many trips have you made over the past month to the hospital/clinic ? ______________
4. Parking (for medical purposes): ($)________________________________________________
5. Meals (during hospital visits/stays): ($)_____________________________________________
6. a. Accommodations (associated with treatment): ($)__________________________________
   1. Please provide the total number of nights spent in accommodation: ___ _________________
   2. What type of accommodations were used: ________________________________________

**HOME SERVICE COSTS**

*For questions 9-11, please indicate in* ***the past month*** *if the following services were received. Please also indicate if a) whether insurance coverage covered all, part, or none of the service, b) the total cost of the service and c) the cost you paid out-of-pocket.* If the service was not received, please mark “0” under the total cost and/or out-of-pocket costs column. (Note: If you had no insurance coverage or financial assistance for these, out-of-pocket costs will be the same as total costs)

|  | Insurance | Total Cost ($) | Out-of-pocket costs ($) (note: will |
| --- | --- | --- | --- |
|  | Coverage |  | be the same as the total cost if you |
|  |  |  | did not receive insurance or other |
|  |  |  | form of coverage) |
| 9. Home Care (e.g. CCAC) | ☐ All |  |  |
|  | ☐ Part |  |  |
| ☐ Not Applicable | ☐ None |  |  |
|  |  |  |  |
| 10. Child Care | ☐ All |  |  |
|  | ☐ Part |  |  |
| ☐ Not Applicable | ☐ None |  |  |
| 11. Domestic Help (e.g. | ☐ All |  |  |
| cooking, cleaning, personal | ☐ Part |  |  |
| support workers) | ☐ None |  |  |
| ☐ Not Applicable |  |  |  |
|  |  |  |  |

**MEDICATIONS, SUPPLEMENTS, OR AIDS**

For questions 12 – 17, please indicate in the past month the costs related to the following medications, supplements, or aids related to your cancer treatment (may list more than 1 per row)? Please also indicate if a) whether insurance coverage covered all, part, or none of the service, b) the total cost of the service and c) the cost you paid out-of-pocket. If the service was not received, please mark “0” under the total cost and/or out-of-pocket costs column. (Note: If you had no insurance coverage or financial assistance for these, out-of-pocket costs will be the same as total costs).

|  | Please  Indicate Type  of  Medication/  Supplement  or Aid | Insurance or  Drug Plan  Coverage | Total Cost  ($) | Out-of-Pocket Costs ($)  (note: will be the same as the total cost if you did not receive insurance or other form of coverage) |
| --- | --- | --- | --- | --- |
|  |  |  |  |  |
|  |  |  |  |  |
|  |  |  |  |  |
|  |  |  |  |  |
|  |  |  |  |  |
| 12. Prescription or over  the counter medications  (eg, chemotherapy  drugs, pain medications) |  | ☐ All  ☐ Part  ☐ None |  |  |
|  |  |  |  |  |
|  |  |  |  |  |
|  |  |  |  |  |
| ☐ Not Applicable |  |  |  |  |
|  |  |  |  |  |
| 13. Complementary, |  | ☐ All  ☐ Part  ☐ None |  |  |
| alternative, or herbal |  |  |  |  |
| medications |  |  |  |  |
| ☐ Not Applicable |  |  |  |  |
| 14. Dietary Supplements  (e.g. isosource,  resource, ensure)  ☐ Not Applicable |  | ☐ All  ☐ Part  ☐ None |  |  |
|  |  |  |  |  |
|  |  |  |  |  |
|  |  |  |  |  |
| 15. Aids or Medical  Devices (e.g. humidified  air device, voice  prosthesis,  tracheostomy tube, arm or leg splint, dressings, bandages, voice prosthesis)  ☐ Not Applicable |  | ☐ All  ☐ Part  ☐ None |  |  |
|  |  |  |  |  |
|  |  |  |  |  |
|  |  |  |  |  |
|  |  |  |  |  |
|  |  |  |  |  |
|  |  |  |  |  |
|  |  |  |  |  |
|  |  |  |  |  |
| 16. Special Garments  ☐ Not Applicable |  | ☐ All  ☐ Part  ☐ None |  |  |
|  |  |  |  |  |
|  |  |  |  |  |
| 17. Vitamins and |  | ☐ All  ☐ Part  ☐ None |  |  |
| Supplements |  |  |  |  |
| ☐ Not Applicable |  |  |  |  |

**ANCILLARY TREATMENT COSTS**

*For questions 18-27, please indicate* ***in the past month*** *the costs for the following services related to your cancer treatment. Please also indicate if a) whether insurance coverage covered all, part, or none of the service, b) the total cost of the service and c) the cost you paid out-of-pocket.* If the service was not received, please mark “0” under the total cost and/or out-of-pocket costs column. (Note: If you had no insurance coverage or financial assistance for these, out-of-pocket costs will be the same as total costs).

|  |  | Insurance | Total Cost ($) | Out-of-Pocket Costs ($) (note: will be the same as total cost if you did not receive insurance) |
| --- | --- | --- | --- | --- |
|  |  | Coverage |  |  |
|  |  |  |  |  |
| 18. Physical Therapy | | ☐ All |  |  |
|  |  | ☐ Part |  |  |
| ☐ Not Applicable | | ☐ None |  |  |
|  |  |  |  |  |
| 19. Occupational Therapy | | ☐ All |  |  |
|  |  | ☐ Part |  |  |
| ☐ Not Applicable | | ☐ None |  |  |
|  |  |  |  |  |
| 20. Speech Language  Pathologist  ☐ Not Applicable | | ☐ All |  |  |
|  |  | ☐ Part |  |  |
|  |  | ☐ None |  |  |
|  |  |  |  |  |
| 21. Dental Visits | | ☐ All |  |  |
|  |  | ☐ Part |  |  |
| ☐ Not Applicable | | ☐ None |  |  |
|  |  |  |  |  |
| 22. Nutritionist | | ☐ All |  |  |
|  |  | ☐ Part |  |  |
| ☐ Not Applicable | | ☐ None |  |  |
|  |  |  |  |  |
| 23. Psychologist | | ☐ All |  |  |
|  |  | ☐ Part |  |  |
| ☐ Not Applicable | | ☐ None |  |  |
|  |  |  |  |  |
| 24. Chiropractor | | ☐ All |  |  |
|  |  | ☐ Part |  |  |
| ☐ Not Applicable | | ☐ None |  |  |
| 25. Massage Therapist | | ☐ All |  |  |
|  |  | ☐ Part |  |  |
| ☐ Not Applicable | | ☐ None |  |  |
|  |  |  |  |  |
| 26. Physician service (not  covered by OHIP, please  specify specialist_______) | | ☐ All |  |  |
|  |  | ☐ Part |  |  |
|  |  | ☐ None |  |  |
| ☐ Not Applicable | |  |  |  |
|  |  |  |  |  |
| 27. Other health care  provider (please specify  specialist) | | ☐ All |  |  |
|  |  | ☐ Part |  |  |
|  |  | ☐ None |  |  |
|  |  |  |  |  |
| ☐ Not Applicable | |  |  |  |
|  |  |  |  |  |

**OTHER COSTS**

If there were additional services that you have used in the past month related to your cancer treatment or supplies/materials that you have purchased, please specify the nature of those costs and the dollar amount associated with them. Please also indicate if a) whether insurance coverage covered all, part, or none of the service, b) the total cost of the service and c) the cost you paid out-of-pocket. If the service was not received, please mark “0” under the total cost and/or out-of-pocket costs column. (note: If you had no insurance coverage or financial assistance for these, out-of-pocket costs will be the same as total costs).

|  | Insurance  Coverage | Total Cost  ($) | Out-of-pocket costs ($)  (note: will be the same as the total cost if you did not receive insurance or other form of coverage) |
| --- | --- | --- | --- |
| 28. Other (please  specify:______________)  ☐ Not Applicable | ☐ All  ☐ Part  ☐ None |  |  |
| 29. Other (please  specify:______________)  ☐ Not Applicable | ☐ All  ☐ Part  ☐ None |  |  |
| 30. Other (please  specify:______________)  ☐ Not Applicable | ☐ All  ☐ Part  ☐ None |  |  |
