# Supplementary material for: Out‐of‐pocket costs associated with head and neck cancer treatment
Source: Cancer Rep (Hoboken). 2021 Aug 24;5(7):e1528. doi: 10.1002/cnr2.1528 (PMC9327650; doi:10.1002/cnr2.1528)
Supplement: Supplementary file 2 — Figure S2 Individual Out of Pocket Costs by Disease Site [file CNR2-5-e1528-s003.docx]

**Supplementary Figure 2. Individual Out of Pocket Costs by Disease Site**


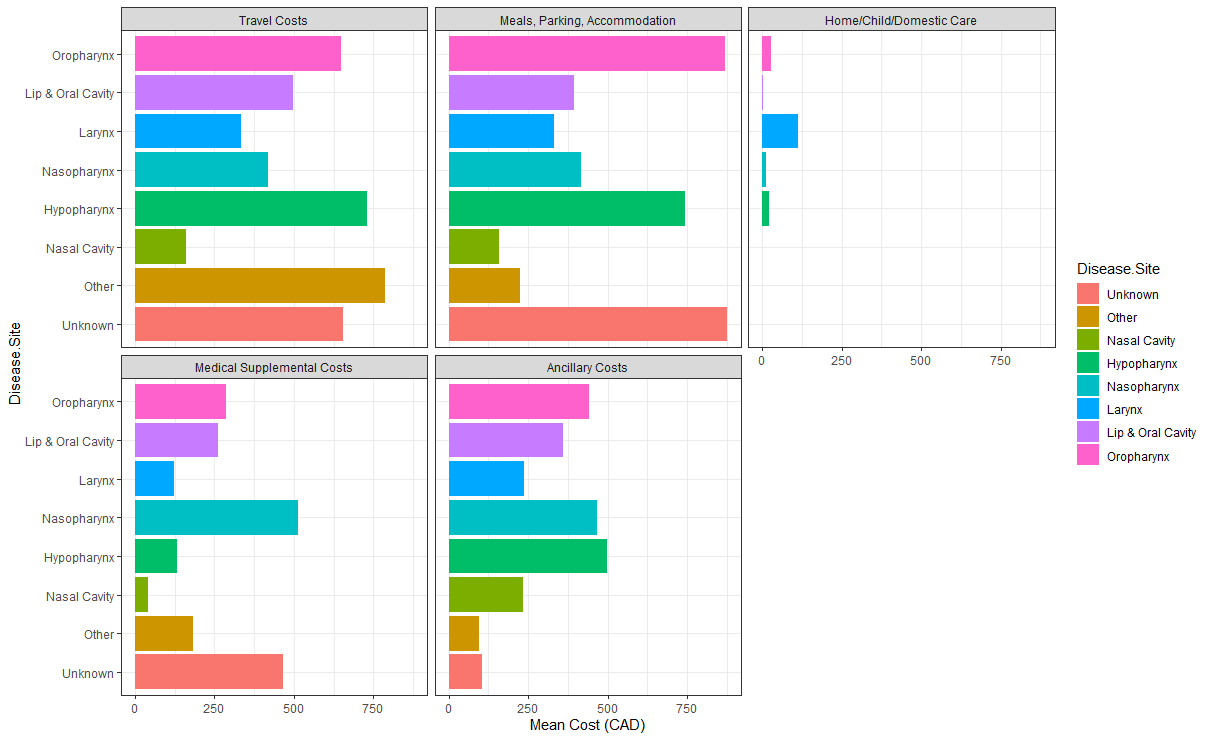


|  | Oropharynx | Lip/Oral Cavity | Larynx | Nasopharynx | Hypopharynx | Nasal Cavity | Other | Unknown |
| --- | --- | --- | --- | --- | --- | --- | --- | --- |
| **N** | 304 | 144 | 99 | 42 | 19 | 11 | 19 | 19 |
| **Treatment Mode** |  |  |  |  |  |  |  |  |
| Surgery Alone | 7 ( 2.3) | 80 (55.6) | 3 ( 3.0) | 0 ( 0.0) | 0 ( 0.0) | 1 ( 9.1) | 4 (21.1) | 1 ( 5.3) |
| Radiation | 128 (42.1) | 3 ( 2.1) | 80 (80.8) | 7 (16.7) | 9 (47.4) | 4 (36.4) | 1 ( 5.3) | 8 (42.1) |
| Surgery + C/RT | 3 ( 1.0) | 57 (39.6) | 7 ( 7.1) | 1 ( 2.4) | 4 (21.1) | 1 ( 9.1) | 13 (68.4) | 0 ( 0.0) |
| Chemoradiation | 166 (54.6) | 4 ( 2.8) | 9 ( 9.1) | 34 (81.0) | 6 (31.6) | 5 (45.5) | 1 ( 5.3) | 10 (52.6) |
| **Clinical Stage** |  |  |  |  |  |  |  |  |
| 0-I | 5 ( 1.6) | 47 (32.9) | 36 (36.4) | 6 (14.3) | 0 ( 0.0) | 6 (54.5) | 6 (33.3) | 0 ( 0.0) |
| II | 13 ( 4.3) | 22 (15.4) | 26 (26.3) | 6 (14.3) | 1 ( 5.3) | 1 ( 9.1) | 3 (16.7) | 0 ( 0.0) |
| III | 36 (11.8) | 20 (14.0) | 19 (19.2) | 14 (33.3) | 3 (15.8) | 1 ( 9.1) | 3 (16.7) | 1 ( 7.1) |
| IV | 250 (82.2) | 54 (37.8) | 18 (18.2) | 16 (38.1) | 15 (78.9) | 3 (27.3) | 6 (33.3) | 13 (92.9) |
| **Age at recruitment** | 61.90 (9.77) | 61.17 (11.32) | 66.22 (10.62) | 55.25 (12.75) | 69.98 (9.61) | 65.93 (9.15) | 59.59 (13.41) | 62.13 (9.48) |
