# Supplementary material for: Out‐of‐pocket costs associated with head and neck cancer treatment
Source: Cancer Rep (Hoboken). 2021 Aug 24;5(7):e1528. doi: 10.1002/cnr2.1528 (PMC9327650; doi:10.1002/cnr2.1528)

**Supplementary Figure 3. Mean and Median Out-of-Pocket Costs over Time**

1. Household Income


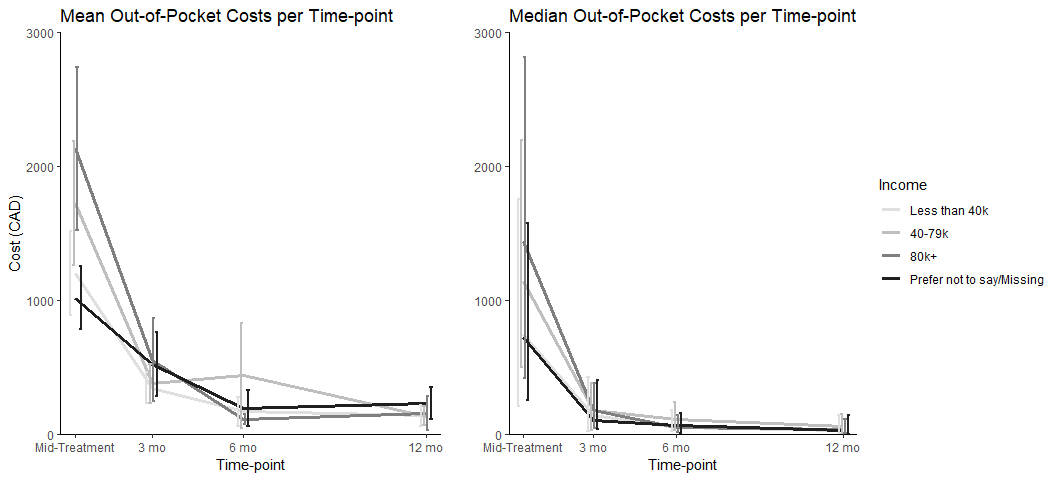


1. Stage of Disease


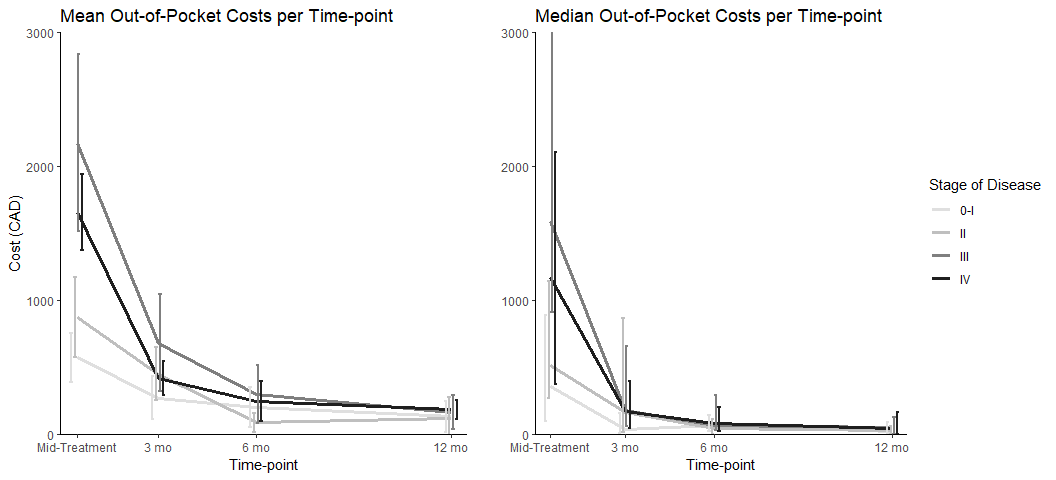


1. Employment Status


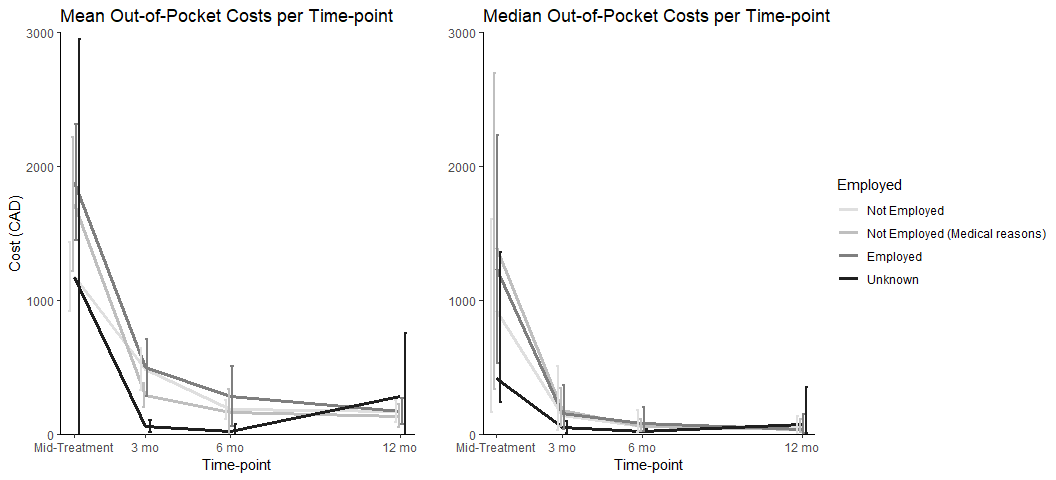


1. Age Group


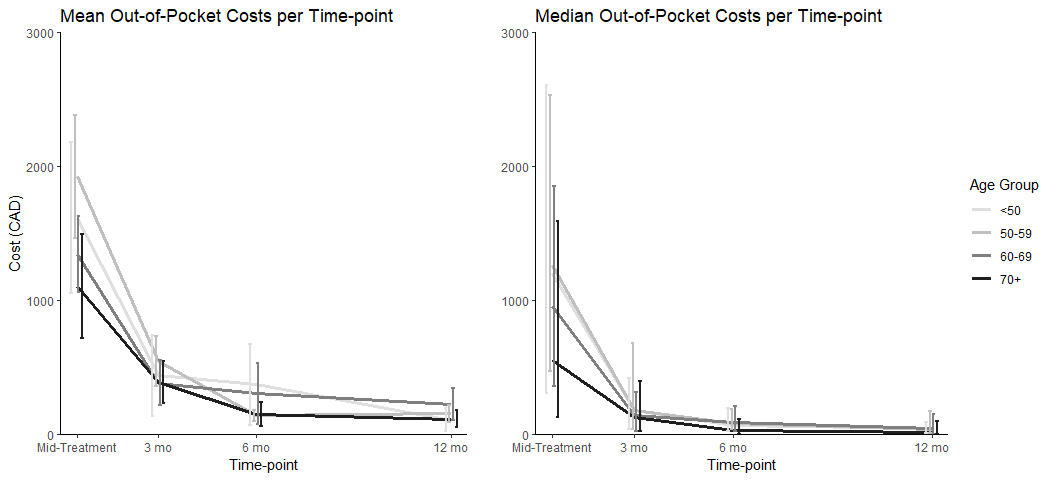


1. Gender


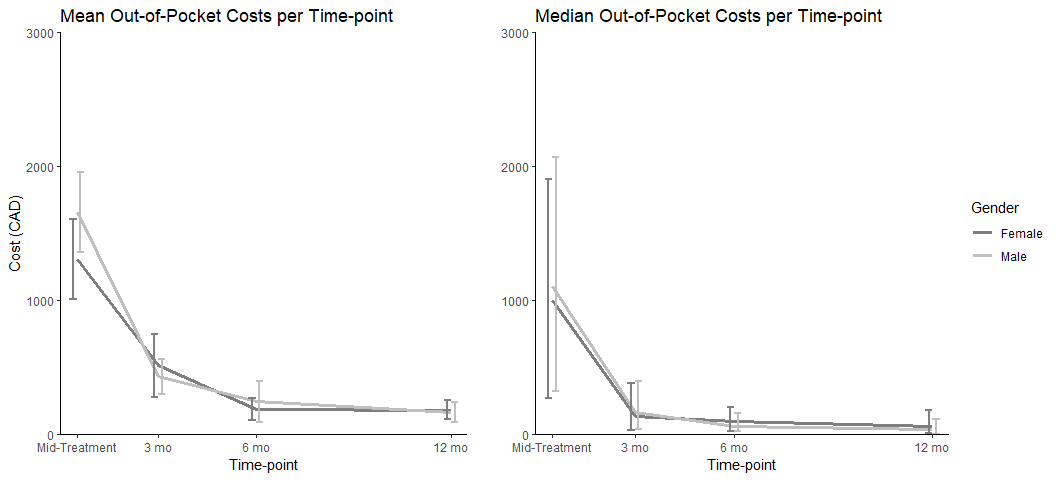

Supplement: Supplementary file 3 — Figure S3 Mean and Median Out‐of‐Pocket Costs over Time [file CNR2-5-e1528-s002.docx]
